# Supplementary material for: A randomized controlled study incorporating an electromechanical gait machine, the Hybrid Assistive Limb, in gait training of patients with severe limitations in walking in the subacute phase after stroke
Source: PLoS One. 2020 Feb 28;15(2):e0229707. doi: 10.1371/journal.pone.0229707 (PMC7048283; doi:10.1371/journal.pone.0229707)
Supplement: S2 Appendix — (PDF) [file pone.0229707.s002.pdf]

Department of Rehabilitation Medicine

Danderyd University Hospital

2013-10-10

Study Plan

Gait training early after stroke – a comparison between the exoskeleton Hybrid Assistive Limb and conventional gait training

Investigators
Anneli Nilsson, Katarina Vreede, Vera Häglund, Disa Sommerfeld, Yoshiyuki Sankai, Hiroaki Kawamoto, Jörgen Borg.

Background
Stroke is a common disorder worldwide and hemiparesis is the most common acute impairment and often impacts on gait function (Duncan 2005, Jorgensen 1995). While motor control is improved in most patients during the months following the ictus, a substantial proportion are left with impaired mobility. Even though several studies indicate that early, intensive training of motor functions may accelerate postacute recovery and improve the final outcome there is a recognised need for more effective treatment in this respect (Langhorne 2009).

Several studies have explored the value of various technologies that are designed to enable more intensive gait training programs (Mehrholtz 2012). While there is evidence that body weight supported gait training on a treadmill may be beneficial early after stroke (Moseley 2005), there is a need for more studies to demonstrate the additional value of training supported by electromechanical devices, which may not only enable more intensive training but also more efficient utilisation of therapist time.

Devices used to support gait training after stroke include treadmill training with or without body weight support (BWS). These devices may be combined with electromechanical “gait machines”, which can allow more reproducible gait movements when compared to when a therapist move the patients legs. Gait machines are often categorized as machines using an end-effector principle and machines that function as exoskeletons (Hesse 2010). Machines based on the end-effector principle use foot plates that move the feet in a controlled gait pattern and allow the operator to adjust many aspects of locomotion, such as speed, stride length and step height. In contrast, exoskeletons are attached to the patient and function as an external skeleton. Exoskeletons for lower extremities have joints matching the patient’s lower limb joints and motors that drive movements over these joints to assist leg movements. Recent reviews conclude that more data is needed with regard to the optimal type of electromechanical device for stroke patients (Merholtz 2012) and that there is a need for further development of concepts and devices as well as their evaluation in clinical trials (Dobkin 2009, Pennycott 2012).

The Hybrid Assistive Limb has been developed at the University of Tsukuba and Cyberdyne Inc in Tsukuba, Japan, in a group headed by professor Sankai (Kawamoto 2009, Kawamoto 2010). The exoskeleton provides support according to the patient’s condition by a control algorithm and supporting devices, where each joint (left and right hip and left and right knee) can be controlled separately. HAL offers a hybrid control algorithm consisting of “Cybernic Voluntary Control”, based on the users voluntary muscle activation and bioelectrical signals, such as by electromyographical input, and a ”Cybernic Autonoumous Control”, based on the users constitution, condition and intensions and using a variety of other signals such as mechanical input. HAL offers a new principle

Study Plan

and allows training early after stroke as soon as postural control is sufficient to allow an upright position. Training by use of HAL may be performed with or without BWS. Our group has recently finalised a study that demonstrates the feasibility and safety of HAL for gait training in patients with impairment early after stroke (Nilsson 2013). This has also been demonstrated after other chronic conditions (Kubota 2013).

Aim and hypotheses of the study
To compare gait function after training with HAL with gait function after conventional gait training as part of an in patient rehabilitation program early after stroke. The primary outcome will be gait performance after the training period and the secondary outcome will be gait performance 6 months after stroke according to the Functional Ambulation Categories. Another outcome will be length of stay in hospital. We hypothesize that training with HAL will improve their gait function significantly more when compared with conventional gait training.

Study design
A randomized controlled study.

Patients
Eligible will be patients living in the Stockholm region and who are admitted to the University Department of Rehabilitation Medicine for inpatient rehabilitation early after stroke from January 2014 until December 2014.

Inclusion criteria: less than eight weeks since stroke; unable to walk independently due to lower extremity paresis (i.e. Functional Ambulation Categories (FAC score 0-1) with or without somato-sensory impairment and with or without spasticity; able to sit on a bench with/without supervision at least five minutes; sufficient postural control to allow upright position in standing with aids and/or manual support; ability to understand training instructions as well as written and oral study information and to express informed consent; body size compatible with the HAL suit.

Exclusion criteria: contracture restricting gait movements at any lower limb joint (hip, knee, ankle); cardiovascular or other somatic condition incompatible with intensive gait training; severe, contagious infections (e. g. with Methicillin Resistant Staphylococcus Aureus (MRSA) or Extended Spectrum Beta-Lactamase (ESBL) bacteria).

Training program and randomisation
Training with HAL will be performed in one session per day, four days per week during four weeks. HAL will be used according to the manufacturer’s manual. Time for each session will be individualised but not exceed 60 minutes/session (effective time). Training with HAL will be performed in combination with body-weight support and treadmill. The training program will be performed by two physiotherapists, who have been trained in the HAL method in Tokyo.

Conventional gait training will be according to current practice, may include use of treadmill and body weight support, and will be documented in the clinical research file (CRF).

Randomisation will be performed by a nurse, who is not otherwise involved in the study according to a block design.

Study Plan

Assessments and blinding
Assessments will be performed by a senior consultant physician (specialist in Rehabilitation Medicine), and by a blinded, registered physiotherapist. All data will be documented by use of standardised forms (paper and pencil) for impairments, activity performance/participation, Quality of Life, and by use of a preformed questionnaire on patients' perceptions and a preformed protocol for adverse events and be kept in individual CRFs.

Standard measures will be used for clinical assessments at baseline, immediately after the training period, and six months after the stroke.

Impairments will be assessed by use of the NIH Stroke Scale (Lyden 1994) for neurological impairments, Fugl-Meyer Scale (FM-LE) (Fugl-Meyer 1975) and the Modified Ashworth Scale (Bohannon and Smith 1987) for the lower extremities.

Activity performance, Participation and Quality of Life will be assessed by use of Functional Ambulation Categories (Holden 1984) (will be performed once a week), 2 minutes walk test in self-preferred speed, Bergs Balance Scale (Berg 1989, 1992), Barthel Index (Mahoney 1965) and EQ5D (Rabin 2001).

Adverse events (such as irritated skin, pain, falls) will be documented continuously in the study protocol according to a specific study form.

Patients' perception of training will be assessed by a questionnaire.

Study documents will be archived at the study site (University Department of Rehabilitation Medicine) according to current routine during 10 years.

Power calculation and statistics
The primary outcome will be the FAC immediately after the training period and at six months after stroke. Based on previous studies of gait recovery early after stroke and of our recent application of HAL early after stroke (Nilsson et al 2013), we estimate that 16 patients per group are needed to demonstrate a clinically meaningful (i.e. one category on the FAC scale) and statistically significant difference between the treatment groups (alpha set at 0.05, power of 80%). Based on our recent experiences, the expected drop out rate is low and we plan to include 36 patients (18/group). The estimated time period for inclusion of all 36 patients is 12 months. Descriptive statistics will be used to describe the two groups at baseline, the Mann Whitney U-test will be used to compare outcomes between treatment groups.

Ethical approval
Submitted October 2013.

Responsibilities
Jörgen Borg serves as principal investigator. Data collection and documentation is delegated to senior consultant Vera Häglund (inclusion procedure, informed consent and neurological examination), physiotherapist Disa Sommerfeld (all other measurements, except Barthel Index), Research Nurse Karolina Krakau (randomization procedure) and physiotherapists Anneli Nilsson and Katarina Vreede (performance of training, data collection during training and reporting of adverse events).

Technical support
Will be provided by Cyberdyne Inc.

References

Duncan PW, Zorowitz R, Bates B, Choi JY, Glasberg JJ, Graham GD, et al. Management of Adult Stroke Rehabilitation Care: a clinical practice guideline. Stroke. 2005 Sep;36(9):e100-43.

Jorgensen HS, Nakayama H, Raaschou HO, Olsen TS. Recovery of walking function in stroke patients: the Copenhagen Stroke Study. Arch Phys Med Rehabil. 1995 Jan;76(1):27-32.

Langhorne P, Coupar F, Pollock A. Motor recovery after stroke: a systematic review. Lancet Neurol. 2009 Aug;8(8):741-54.

Mehrholz J, Pohl M. Electromechanical-assisted gait training after stroke: a systematic review comparing end-effector and exoskeleton devices. J Rehabil Med. 2012 Mar;44(3):193-9.

Moseley AM, Stark A, Cameron ID, Pollock A. Treadmill training and body weight support for walking after stroke. Cochrane Database Syst Rev. 2005;(4):CD002840.

Hesse S, Waldner A, Tomelleri C. Innovative gait robot for the repetitive practice of floor walking and stair climbing up and down in stroke patients. J Neuroeng Rehabil. 2010;7:30.

Dobkin BH. Motor rehabilitation after stroke, traumatic brain, and spinal cord injury: common denominators within recent clinical trials. Curr Opin Neurol. 2009 Dec;22(6):563-9.

Pennycott A, Wyss D, Vallery H, Klamroth-Marganska V, Riener R. Towards more effective robotic gait training for stroke rehabilitation: a review. J Neuroeng Rehabil. 2012;9:65.

Kawamoto H, Hayashi T, Sakurai T, Eguchi K, Sankai Y, “Development of Single Leg Version of HAL for Hemiplegia”, Proceedings of 31st Annual International Conference of the IEEE Engineering in Medicine and Biology Society, pp. 5038-5043, 2009

Kawamoto H, Taal S, Niniss H, Hayashi T, Kamibayashi K, Eguchi K, Sankai Y, “Voluntary Motion Support Control of Robot Suit HAL Triggered by Bioelectrical Signal for Hemiplegia”, Proceedings of 32nd Annual International Conference of the IEEE Engineering in Medicine and Biology Society, pp. 462-466, 2010

Nilsson A, Skough Vreede K, Häglund V, Kawamoto H, Sankai Y, Borg J. Gait training early after stroke with a new exoskeleton – the Hybrid Assistive Limb: A pilot study of safety and feasibility. Submitted to Neurorehabilitation and neural repair, Sept 2013.

Kubota S, Nakata Y, Eguchi K, Kawamoto H, Kamibayashi K, Sakane M, et al. Feasibility of rehabilitation training with a newly developed wearable robot for patients with limited mobility. Arch Phys Med Rehabil. 2013 Jun;94(6):1080-7.

Lyden P, Brott T, Tilley B, Welch KM, Mascha EJ, Levine S, et al. Improved reliability of the NIH Stroke Scale using video training. NINDS TPA Stroke Study Group. Stroke. 1994 Nov;25(11):2220-6.

Fugl-Meyer AR, Jaasko L, Leyman I, Olsson S, Steglind S. The post-stroke hemiplegic patient. 1. a method for evaluation of physical performance. Scand J Rehabil Med. 1975;7(1):13-31.

Study Plan

Bohannon RW, Smith MB. Phys Ther. Interrater reliability of a modified Ashworth scale of muscle spasticity. 1987 Feb;67(2):206-7.

Holden MK, Gill KM, Magliozzi MR, Nathan J, Piehl-Baker L. Clinical gait assessment in the neurologically impaired. Reliability and meaningfulness. Phys Ther. 1984 Jan;64(1):35-40.

Berg K W-DS, Williams J.I., Gayton D. Measuring balance in the enderly: preliminary development of an instrument. Physiother Can. 1989;41(6):304-11.

Berg K MB, Williams J, Holliday P, Wood-Dauphinée S. Clinical and laboratory Measures of Postrural Balance in an Elderly Population. Arch Phys Med Rehabil. 1992;73:1073-80.

Mahoney FI, Barthel DW. Functional Evaluation: The Barthel Index. Md State Med J. 1965 Feb;14:61-5.

Rabin R, de Charro F. EQ-5D: a measure of health status from the EuroQol Group. Ann Med. 2001 Jul;33(5):337-43.
