# Supplementary material for: A randomized controlled study incorporating an electromechanical gait machine, the Hybrid Assistive Limb, in gait training of patients with severe limitations in walking in the subacute phase after stroke
Source: PLoS One. 2020 Feb 28;15(2):e0229707. doi: 10.1371/journal.pone.0229707 (PMC7048283; doi:10.1371/journal.pone.0229707)
Supplement: Attachment — Submitted filename: Response to Reviewers.docx [file pone.0229707.s004.docx]

**Response to Reviewers**

We are grateful for the constructive comments and questions from the editor and reviewers and have considered all points in the revised version. Please find our point-to-point responses and attached manuscript with and without track changes.

Response to Journal Requirements

1. We have now updated the manuscript to meet PLOS ONE's style requirements.
2. We have changed the following text in the Methods, Design section, to clarify the recruitment period and the study registration procedure. Now to read:

*This prospective, randomized, open labeled, blinded evaluation (PROBE) study was conducted at the University Department of Rehabilitation Medicine at Danderyd Hospital, Stockholm, Sweden, admitting patients aged 18-67 years with moderate to severe acquired brain injury. The recruitment period lasted between February 2014 and December 2016, with the last follow-up performed in May 2017. All participants in this study expressed informed consent. The study was approved by the Regional Ethical Committee of Stockholm on November 13, 2013 (2013/1807-31/2) and was performed in compliance with GCP and the Declaration of Helsinki. The study was registered at ClinicalTrials.gov Identifier: NCT02410915,* [*https://clinicaltrials.gov/ct2/show/NCT02410915*](https://clinicaltrials.gov/ct2/show/NCT02410915)*. Registration was published in April 2015 after start of enrollment since registration was not part of the research routine at our department prior to this date. The authors confirm that all ongoing and related trials by our study group for this intervention are registered.*

Response to Reviewer #1

Thank you for relevant comments and questions. The responses for each query are mentioned below following the specific query.

**1. The primary outcome analysis seems to be based on Friedman's test or ordinal regression (undefined: please include the specific model you are using), but this is not stated. It is not stated why "1" is a clinically relevant difference. It is not stated why "1" is a reasonable S.D. It is not stated what is being tested and how (if it's Friedman's test you cannot use the formula for a t-test). With such a small sample size, it is unlikely that the central limit theorem will take care of the normality, so this needs to be stated much more clearly.**

We apologize for causing any confusion. For information about statistical methods used we refer to the Methods, Statistic section. We also added clarifying information regarding this in the text (see line 364 and 394 and 383) and in Table 3 (table legend).

We added the following text to the Methods, Power calculation and sample size section:

*The power calculation was based on available data from previous studies in this area [17, 30] using the FAC score. The score has been found to have good responsiveness and concurrent validity of each step along the scale in relation to functional mobility, walking distance and walking speed [31].*

**2. In the conclusion section it should be stated whether the assumptions of the same size computation (e.g., S.D., effect size) were realized.**

The following in described in the Limitations section:

*Because the sample size was small, caution must be applied when interpreting our results. Power calculation was performed for our primary outcome; however, two patients were lost to the 6 months follow up, which reduced the power. In addition, between-group differences in the primary outcome were less and the variance was greater than expected.*

We added the following text in the Conclusion section, now to read:

*Despite the finding that incorporated HAL training was not superior to CGT in this small study sample, our results indicate that HAL training was well tolerated and might provide a way of achieving more intensive gait training, as illustrated by the longer walking distances achieved during the training sessions. The results should be interpreted with caution considering that the sample size was small, and that between-group differences were less and variance greater than expected.*

**3. Your tables have a bunch a stars indicated significance at 0.05 but you have not made any attempt at adjustment for multiple testing. Unless these are purely exploratory hypotheses, you have not preserved your type I error rate.**

Thank you for this comment. We have again consulted a statistician regarding adjustment for multiple testing. In the revised version we applied this adjustment (see Table 3) with the new p-value of 0.006 (previous p-value divided by number of tests for each outcome = 0.05/9).

We added the following text in the Methods, Statistics section:

*In case of multiple testing the p-value was adjusted according to Bonferroni based on the number of tests for each outcome.*

We also added the following text in Table 3 (table legend):

** Indicates significant difference after adjustment for multiple testing (p < 0.006)*

Response to Reviewer #2

Thank you for relevant comments and questions. The responses for each query are mentioned below following the specific query.

**Abstract**

**1. The abstract conclusion should relate to the main aim of the study. Eg there were no differences between groups – especially considering the extra time and resources the HAL training required.**

We clarified this in the abstract conclusion. Now to read:

*This study found no difference between groups for any outcomes despite the extra resources required for the HAL training, but highlights the substantial improvements in walking seen when evidence-based rehabilitation is provided to patients, with severe limitations in walking in the subacute stage after stroke. In future studies potential subgroups of patients who will benefit the most from electromechanically assisted gait training as well as positive cardiovascular effects related to the longer walking distances enabled by this training should be explored.*

Note that small additional changes were made in the abstract to meat the requirement of maximum 300 words.

**Introduction**

**2. The introduction contains information that would be better described in the methods. Eg lines 96-105 and Figure 1.**

Part of the suggested lines were moved, and the following text was added/changed in the Methods, Gait training with HAL section:

*Patients used the handrail on the non-paretic side for support. During each session, output was adjusted via a control unit by which the therapist adapted the level of assistance over the hip and knee joint. The settings were individually adapted as the training progressed and optimized based on continuous observational gait analysis [22] in order to achieve a gait pattern as close to normal gait as possible (i.e. avoiding compensatory movements such as circumduction).*

Figure 1 was moved to the Methods, Gait training with HAL section as suggested.

**3. More description of the prior clinical trial could be provided in the introduction in relations to the findings and need for this RCT.**

We added/changes the following text in the Introduction section, now to read:

*The Hybrid Assistive Limb has been found feasible for gait training in patients with lower extremity paresis in the subacute stage after stroke, as well as in the long term phase [16]. Our piloting feasibility study [17], preceding the current study, found improvements in motor function, walking independence- and speed, balance, and activities of daily living performance after HAL training. The median (range) FAC-score improved from 0 (0–2) to 1.5 (1–4) from baseline to after the intervention. Later, additional studies have indicated beneficial effects on gait function and independence in walking [18-20] after HAL training, but the data do not allow any firm conclusions. Further, most previous studies of HAL were single group studies (without control treatment), why there was a need for blinded, randomized controlled trials.*

**4. Short and long term should be defined**

This was removed. The sentence is now reading:

*Thus, the overall aim for the study was to evaluate the potential benefit on functioning and disability, of incorporating HAL training, compared to conventional gait training (CGT) only, as part of an inpatient rehabilitation program in the subacute stage after stroke.*

**Methods**

**5. There was a large number of participants that were ineligible. Could reasons for exclsuion be provided?**

The following sentence was added to the Results, Patient characteristics section:

*Most common reasons for exclusion were diagnoses other than stroke and/or a FAC score >1.*

**6. What was the body size limit for the equipment (line 156)**

Response to comment: Overall, the body weight should not exceed 100kg and the height should be between 150-195 cm. However, even if the weight and/or height of a patient exceeded these ranges, a wearers upper leg length, lower leg length, and hip width might still allow use of HAL, why we chose not to specify body size in the manuscript.

**7. The randomization procedure is not sufficient described**

We changed the following sentence in the Methods, Randomization section, now to read:

*A nurse, not otherwise involved in the study manually randomized the patients according to a block design to either incorporated HAL training (HAL group) or CGT only (CONV group). For logistic reasons, randomization was performed in blocks of four (e.g. HAL, HAL, CONV, CONV or HAL, CONV, HAL, CONV etc.) to enable consecutive inclusion.*

**8. It is not necessary to statistically test the difference in the table of characteristics (Table 2). The sample size calculations are not designed to test this.**

This column (with p-values) was removed from Table 2.

**Results**

**9. The text is quite repetitive of what is in the table and therefore could be shortened.**

The results have been shortened accordingly. Please see the attached manuscript file.

**Discussion**

**10. The discussion is very long and would benefit from being shortened with greater focus on the main results of this study eg paragraph 2 should discuss the main results and why there might not have been differences between groups. The HAL group received more therapy time and sessions (as they also received conventional training) and HAL training required mostly 2 therapists. Despite this there were no significant differences between groups.**

The discussion has been shortened and restructured accordingly. Please see the attached manuscript file

**11. In the HAL group HAL training resulted in greater distance than in conventional training. Interestingly the HAL group walked on average 50% less during conventional training than the conventional group. Perhaps mention should also be made of this?**

We clarified this in the Discussion section line 556, now to read:

*However, as presented above, the number of CGT sessions were significantly fewer and although non-significant the distances walked during the CGT sessions shorter in the HAL group.*

**Conclusion**

**12. The conclusion should better reflect the results - that there were no difference between groups for any outcomes despite the extra sessions, time and resources required for the HAL training. An equivalence trial was not carried out – therefore the statement regarding equal improvement should be removed.**

We changed the following sentence in the conclusion, now reading:

*This study found no difference between groups for any outcomes despite the extra resources required for the HAL training. Notably, substantial improvements were observed after 4 weeks of incorporated HAL training and conventional gait training only, with regard to gait function, mobility, and self-care in younger hemi-paretic patients with severely impaired mobility in the subacute stage after stroke.*
