# Supplementary material for: A randomized controlled study incorporating an electromechanical gait machine, the Hybrid Assistive Limb, in gait training of patients with severe limitations in walking in the subacute phase after stroke
Source: PLoS One. 2020 Feb 28;15(2):e0229707. doi: 10.1371/journal.pone.0229707 (PMC7048283; doi:10.1371/journal.pone.0229707)
Supplement: Attachment — Submitted filename: Response to Reviewers.docx [file pone.0229707.s006.docx]

**Response to Reviewers/Editor**

We are grateful for the constructive comments from the editor and have considered all points in the revised version. Please find our responses and attached manuscript with and without track changes.

Response to Editor Comments:

**The columns with p-values for within-group differences were removed from Table 2.**

**The abstract, results and discussion were changed to focus on between-group comparisons (all within-group comparisons were removed). In addition, the statements about additional outcomes, such as the longer walking distances achieved, was removed from the conclusion.**

**In the Abstract now to read:** *No significant between-group differences were found regarding any primary or secondary outcomes. At 6 months, two thirds of all patients were independent in walking. Prediction of independent walking at 6 months was not influenced by treatment group, but by age (OR 0.848, CI 0.719–0.998, p=0.048).*

**In the Discussion now to read:** *The main finding of this PROBE study was that there was no significant difference after 4 weeks of HAL training with regard to independence in walking, movement function, self-selected walking speed, balance, or self-care when added to conventional training in the subacute phase for patients with severe limitations in walking 5 weeks after stroke. The odds of being independent in walking at 6 months post stroke were influenced by age, but not by treatment. These results indicate that incorporating HAL training does not influence the beneficial outcome seen in these younger patients who received evidence-based, CGT in a specialized neurorehabilitation setting due to severely limited walking ability after stroke.*

**In the Conclusion now to read:** *This study found no difference between groups for any outcomes after the intervention or at 6 months post stroke, despite the extra resources required for the HAL training. In these younger patients with hemiparesis and severe limitations in walking in the subacute stage after stroke independence in walking at 6 months was not related to group allocation but to a younger age. The results should be interpreted with caution considering that the sample size was small, and that between-group differences were less and variance greater than expected. The findings suggest that further explorations of who in the heterogeneous stroke population will benefit the most from EAGT should be addressed in future studies.*

**Table 1 and 4 were removed and described in the text.**

**Additional information: In the revised version we do not apply an adjustment for multiple comparisons (according to Bonferroni) since the within-group calculations were removed. The p-value used is thus 0.05. Note that even if adjustment for multiple testing would be applied, this would not change the results.**
